# The Efficacy of Cannabis in Oncology Patient Care and Its Anti-Tumor Effects

**DOI:** 10.3390/cancers16162909

**Published:** 2024-08-21

**Authors:** Walid Shalata, Omar Abu Saleh, Lena Tourkey, Sondos Shalata, Ala Eddin Neime, Ali Abu Juma’a, Arina Soklakova, Lama Tourkey, Ashraf Abu Jama, Alexander Yakobson

**Affiliations:** 1 The Legacy Heritage Center and Dr. Larry Norton Institute, Soroka Medical Center, Beer Sheva 84105, Israel; 2 Medical School for International Health, Faculty of Health Sciences, Ben Gurion University of the Negev, Beer Sheva 84105, Israel; 3 Department of Dermatology and Venereology, Emek Medical Centre, Afula 18341, Israel; 4 Nutrition Unit, Galilee Medical Center, Nahariya 22000, Israel

**Keywords:** cannabis, cancer patients, cannabis and oncologic patients, endocannabinoid system, cannabis consumption, anti-tumor effect of cannabis

## Abstract

**Simple Summary:**

Cancer is a major disease and a leading cause of death worldwide. Improving treatment and management strategies for cancer is critical. This article explores cannabis and its pharmacological properties as a promising tool in cancer care, especially in easing symptoms like appetite loss, pain, nausea, vomiting, and insomnia. Moreover, it examines the anti-tumor properties of cannabis, highlighting that, although some evidence suggests benefits, more research is necessary to confirm these effects. The article addresses the evidence concerning the clinical challenges of using cannabis, such as its psychoactive effects, and potential side effects. The article aims to clarify the current understanding of cannabis use in cancer care, helping healthcare professionals and patients make better-informed decisions and improve treatment outcomes.

**Abstract:**

As the legalization of medical cannabis expands across several countries, interest in its potential advantages among cancer patients and caregivers is burgeoning. However, patients seeking to integrate cannabis into their treatment often encounter frustration when their oncologists lack adequate information to offer guidance. This knowledge gap is exacerbated by the scarcity of published literature on the benefits of medical cannabis, leaving oncologists reliant on evidence-based data disheartened. This comprehensive narrative article, tailored for both clinicians and patients, endeavors to bridge these informational voids. It synthesizes cannabis history, pharmacology, and physiology and focuses on addressing various symptoms prevalent in cancer care, including insomnia, nausea and vomiting, appetite issues, pain management, and potential anti-cancer effects. Furthermore, by delving into the potential mechanisms of action and exploring their relevance in cancer treatment, this article aims to shed light on the potential benefits and effects of cannabis in oncology.

## 1. Introduction

Over one hundred years ago, the endocannabinoid system emerged as a fundamental aspect of human physiology. Subsequently, there has been a more systematic exploration of the medicinal uses and properties of cannabis [1]. Over the last few decades in the medical world, there has been growing interest in utilizing cannabinoids for symptom management in patients with cancer or HIV, as well as in conditions such as epilepsy, Tourette syndrome, spasticity, and digestive disorders [2,3]. The controversies surrounding the legalization of cannabis for recreational purposes hinder the approval process for its medical applications, reminiscent of the debates in the 1980s that hindered the adoption of opioid-based treatments for cancer pain. These debates often reflect political agendas rather than strictly medical considerations. Regarding the medical use of cannabis, two opposing viewpoints emerge: one is supportive, sometimes regardless of clinical evidence, while the other is conservative, driven by preconceptions and concerns [2,3,4]. Cannabis is comprised of over 500 compounds, with at least 100 identified as cannabinoids, known as phytocannabinoids, which originate from trichomes found in the female plants of Cannabis sativa. Among these, the most prevalent are Δ-9-tetrahydrocannabinol (Δ9-THC), which is responsible for psychoactive effects, and cannabidiol, which lacks psychoactivity. Additionally, cannabis contains flavonoids and terpenes. These discoveries have led to the identification of cannabinoid receptor 1, predominantly found in the central nervous system (CNS), and cannabinoid receptor 2, primarily expressed in immune cells. Furthermore, cannabinoids interact with these receptors, on immune and tumor cells, leading to various anti-cancer effects. These include inducing cancer cell death, inhibiting tumor growth, and suppressing metastasis. Cannabinoids also influence immune cells within the tumor microenvironment, a critical factor in cancer progression and spread [5]. Notably, CB1 and CB2 agonists (ACEA and JWH-133) selectively inhibit VEGF-A production, a potent angiogenic and vasoactive mediator, from LPS-activated human polymorphonuclear neutrophils, without altering the release of other angiogenic factors such as CXCL8 and HGF; consequently, this inhibition results in reduced angiogenesis and endothelial permeability, which are critical in the pathophysiology of sepsis and cancer. Therefore, understanding the role of CB1 and CB2 receptors on the immune cells could lead to the development of targeted cancer therapies [6]. The discoveries have also revealed the existence of endogenous ligands such as anandamide (AEA) and 2-arachidonoylglycerol (2-AG). Subsequently, the enzymes responsible for the synthesis and degradation of these ligands have been identified, including N-acyltransferase (NAT) and N-acyl-phosphatidylethanolamine-hydrolyzing phospholipase (NAPE-PLD) for AEA, and fatty acid amide hydrolase (FAAH) for AEA degradation. For 2-AG, the enzymes involved in synthesis include diacylglycerol lipase (DAGLa/b), while degradation is regulated by monoacylglycerol lipase (MAGL). Collectively, these components constitute the endocannabinoid system (ECS) [7,8,9,10,11]. Comprehending drug interactions poses a fundamental but intricate pharmacological concern, particularly accentuated in the field of oncology. This complexity arises from the typically narrow therapeutic window and the potential for severe toxicity associated with drugs frequently administered to vulnerable patients with extensive pre-treatment histories. Interactions between drugs can stem from pharmacokinetic, pharmacodynamic, or biological factors, leading to a spectrum of outcomes, ranging from diminished or heightened therapeutic effects to increased risk of adverse reactions. Despite significant research and development efforts, most anti-cancer therapies are associated with severe adverse effects, prompting the widespread use of cannabis plant-based products to mitigate them. Recent studies have shown that approximately 60–70% of cancer patients incorporate cannabis products into their ongoing therapy to alleviate adverse effects. Cannabis products are primarily consumed through inhalation, ingestion, or topical application, with forms such as edibles, liquids, or smoked/vaporized cannabis being prevalent [12,13,14,15].

This article aims to provide a comprehensive review of the literature concerning the anti-cancer effects of both plant-derived and synthetic cannabinoids. By delving into their potential mechanisms of action and exploring their role in cancer treatment, we seek to enhance our understanding of these compounds. Additionally, we examine the current legislative landscape surrounding the medical and therapeutic use of cannabinoids.

## 2. Materials and Methods

Extensive searches were conducted on PubMed, Scopus, and Web of Science from their inceptions to April 2024 to pinpoint clinical trials and review articles evaluating the efficacy of cannabis for oncology patients. The search terms included “cannabis”, “cancer patients”, “oncology”, “cancer treatment”, “endocannabinoid system”, “history of cannabinoids”, “cannabis and oncologic patients”, “pharmacology of cannabis”, “physiology of cannabis”, “post-harvest cannabis”, “cannabis consumption”, “anti-tumor effect of cannabis”, and “cannabis in managing cancer symptoms”. The inclusion criteria for the works discussed in the study were articles that provided accessible and relevant data on the use of cannabis in oncology, particularly focusing on its anti-tumor effects, its role in symptom management, and its pharmacological and therapeutic mechanisms. Exclusion criteria were likely articles not directly related to cancer or cannabis, as well as studies that were not accessible.

## 3. History of Cannabinoids

Cannabis sativa serves as the primary and earliest known source of cannabinoids. It has a rich history of medicinal usage worldwide, dating back thousands of years. For instance, historical records from China dating back to the 28th century BCE, credited to Emperor Shen Nung, describe cannabis as being employed to address various health concerns, such as issues affecting reproductive organs of women, chronic rheumatic pain, malaria, and gastrointestinal problems like constipation. Moreover, its therapeutic use was revived in the mid-19th century by Irish physician William B. O’Shaughnessy and French psychiatrist Jacques-Joseph Moreau [16,17,18]. There is documented evidence showing the beneficial effects of cannabis preparations on pain, vomiting, convulsions, rheumatism, tetanus, and cognitive function. By 1851, cannabis had gained recognition as a medicinal substance in the United States pharmacopeia, available in forms such as tinctures, extracts, and resins. However, at the turn of the 20th century, its medicinal use declined due to increased recreational use, concerns about abuse potential, variability in herbal material quality, unidentified active compounds, and the introduction of alternative medications with established efficacies for similar symptoms. In 1941, due to mounting legal restrictions, cannabis was classified alongside other illicit drugs and removed from the American pharmacopeia. Consequently, research into the medicinal applications of cannabis slowed significantly for over a half-century [16,17,18,19].

## 4. Pharmacology and Physiology of Cannabis

**Figure 1:**
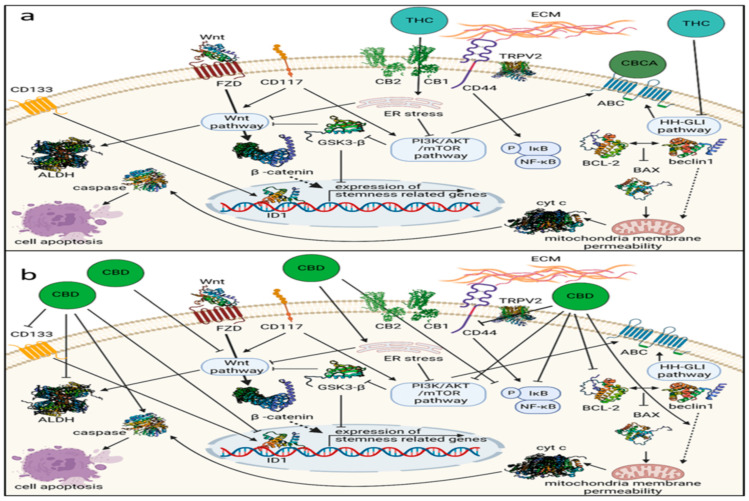
Phytocannabinoids pathways and mechanisms like THC and CBCA, (**a**) along with CBD, (**b**) impact several genetic pathways and mechanisms linked to the ovarian cancer stem cell state. Receptor involvement in activity is indicated where suggested. Key components include ABC (ATP-binding cassette transporter), ALDH (aldehyde dehydrogenase), BCL-2 (B-cell lymphoma-2 activity), CB1 (cannabinoid receptor type 1), CB2 (cannabinoid receptor type 2), CBCA (cannabichromenic acid), CBD (cannabidiol), CDs (clusters of differentiation), cyt c (cytochrome c), ECM (extracellular matrix), ER stress (endoplasmic reticulum stress), FZD (Wnt frizzled receptor), HH-GLI (Hedgehog-GLI), ID1 (inhibitor of DNA binding), THC (Δ9-trans-tetrahydrocannabinol), and TRPV2 (transient receptor potential cation channel subfamily V member 2) [28].

Phytocannabinoids are naturally occurring compounds with a limited distribution in nature, found in various taxonomic groups such as liverworts, fungi, and plants. Traditionally, phytocannabinoids have been primarily associated with cannabis species and related plant analogs. Cannabis sativa, for example, contains over 500 chemical compounds, including well-known cannabinoids like Δ9-THC and cannabidiol. Cannabinoid receptors, as transmembrane proteins, facilitate the effects of cannabinoids [20,21,22]. There are two primary cannabinoid receptors, known as cannabinoid receptor 1 and cannabinoid receptor 2, belonging to the class A family of G-protein-coupled receptors. These receptors consist of extracellular regions containing glycosylated amino-terminals, as well as intracellular regions containing carboxy-terminal domains. They are connected by seven transmembrane domains, along with three extracellular loops and three intracellular loops. Cannabinoids exert their pharmacological effects through the endogenous cannabinoid system, which includes CB1 and CB2 receptors. In addition to binding to cannabinoid receptors, cannabinoids also interact with non-cannabinoid receptor 2/non-cannabinoid receptor 2 receptors like G-protein-coupled receptor 55 and transient receptor potential channels to elicit their effects (Figure 1). The endogenous cannabinoid system regulates a wide range of physiological functions, including immunological and neurological processes. Furthermore, cannabinoids play roles in various physiological conditions and processes such as mood regulation and anxiety disorders, appetite modulation, Parkinson’s disease, and memory function by its receptors that are present in tissues throughout the body, including both central and peripheral locations [20,21,22,23,24,25,26,27].

Phytocannabinoids, which are biosynthesized by specific enzymes, are primarily responsible for the therapeutic properties of cannabis. The composition and concentration of these compounds, such as cannabigerol, Δ9-THC, CBD, and cannabichromene (CBC), vary depending on tissue type, age, variety, growth conditions, and harvest time. Postharvest, these compounds can degrade, for example as Δ9-tetrahydrocannabinolic acid (Δ9-THCA) converting into the psychoactive Δ9-THC through heat-induced decarboxylation [23]. Furthermore, terpenes and terpenoids in *Cannabis sativa* have diverse biological activities, such as anti-fungal, anti-viral, and anti-cancer properties, and are crucial for the plant’s aroma. The chemical composition of these compounds can change due to environmental factors, distillation, and storage conditions. During storage, especially under poor conditions or prolonged exposure to air and UV light, degradation and oxidation can alter the efficacy and safety of essential oils, potentially transforming terpenes into allergens and losing volatile components [24]. The “entourage effect” emphasizes the enhanced benefits of using cannabinoids and terpenes together, highlighting the complex therapeutic potential of cannabis [25]. Therefore, chemical modifications that happen during post-harvest and usage processes can significantly alter the chemical composition and therapeutic profiles of cannabis by influencing the chemical profiles of cannabinoids and terpenes.

## 5. Methods of Cannabis Consumption

### 5.1. Inhalation Use

Inhalation remains the predominant means of cannabis intake across the United States and globally. This method encompasses smoking, wherein the dried flower is ignited, and its released components are inhaled. Smoking can take various forms, such as rolled cigarette joints or pipe bongs. Vaporization, on the other hand, involves heating the plant to a temperature that releases its active ingredients as inhalable vapor without combustion. While the long-term effects of vaporized cannabis inhalation are still not fully understood previously, a meta-analysis suggested minimal impact on pulmonary function in the short term. However, vaporizers utilize concentrated plant oil, sometimes containing up to 90% Δ9-THC, which could lead to severe side effects for inexperienced users. Inhaling such high Δ9-THC concentrations, whether through smoking or vaporization, may heighten the risk of arrhythmia or myocardial infarction in susceptible individuals. Common side effects associated with inhalation include a sore throat, irritation of the oral mucosa, and coughing. An advantage of inhalation is its rapid onset of action, particularly beneficial when nausea is a prominent symptom, and the ease of dose titration, which reduces the likelihood of overconsumption [29,30,31,32,33].

### 5.2. Oral Use

The trend toward oral cannabis consumption is on the rise, with various innovative methods emerging. Recently, there has been a surge in the development of cannabis-infused products, particularly beverages and food items. Additionally, sublingual ingestion methods, like dissolvable strips, sprays, lozenges, and tinctures, are also gaining attention. However, a major challenge with oral or sublingual ingestion lies in its poor pharmacokinetics. The lipophilic nature of the bioactive compounds contributes to the challenge of achieving high bioavailability, which typically ranges between 6% and 25%. However, absorption can be unpredictable, with delays or variations influenced by stomach contents. This variability makes it challenging to titrate doses effectively and increases the risk of overconsumption, particularly with high-Δ9-THC products. Patients may mistakenly assume they need more due to the delayed onset of effects, leading to potential adverse reactions such as anxiety, nausea, paranoia, short-term psychosis, and disorientation. Another less common but recognized method is sublingual administration, which holds the potential for enhancing bioavailability and absorption. An example of this is Sativex (nabiximols), the only plant-based cannabinoid medication approved for medical use in multiple countries, including parts of Europe and Canada. It is administered as a sublingual spray. Although its onset time is similar to traditional oral consumption, certain studies indicate a potentially quicker onset with sublingual administration [34,35,36].

### 5.3. Topical Use

Another less conventional method of cannabis consumption involves topical application in the forms of patches, salves, lotions, and oils. This approach offers the advantage of providing a sustained drug release over an extended period while minimizing the risk of adverse effects associated with high peak concentrations due to limited systemic absorption. Topical administration is particularly well-suited for addressing localized symptoms, such as those seen in dermatologic conditions and arthritis. Nonetheless, it is important to note that local skin irritation may occur, and the absorption characteristics of both the cannabis preparation and any additives may not be fully understood. Despite these considerations, topical use remains popular among new users and older adults seeking relief from symptoms without experiencing the intoxicating effects of cannabinoids [33,37].

## 6. The Anti-Tumor Effect of Cannabis

**Figure 2:**
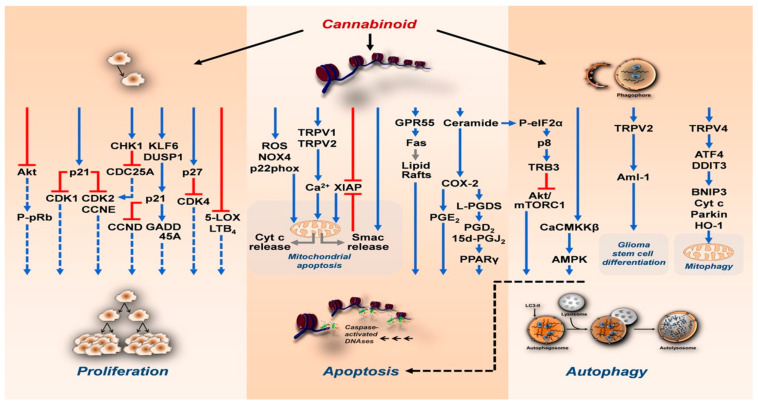
Cannabinoids’ mechanisms on cancer cells [48].

Cannabinoids have emerged as valuable agents in cancer therapy, demonstrating significant palliative effects in managing symptoms like nausea, vomiting, pain, and loss of appetite. Beyond symptom relief, cannabinoids exhibit promising anti-tumor actions by modulating intracellular signaling pathways involved in cancer progression. Initial findings suggest that Δ9-THC inhibits the growth of lung adenocarcinoma cells in vitro and murine models post-oral administration, directly inhibiting cell proliferation and promoting apoptosis. Moreover, cannabinoids interfere with processes like angiogenesis, invasion, and metastasis. Multiple studies across various cancer cell lines and animal tumor models support these findings. Endocannabinoids like N-arachidonoyl ethanolamine-anandamide have shown anti-proliferative effects against other carcinomas by downregulating the expression of the epidermal growth factor receptor and increasing ceramide production [16,38,39,40,41,42,43,44,45,46,47]. Phytocannabinoids like Δ9-THC have been found to reduce tumor proliferation, inhibit angiogenesis, and induce apoptosis in breast cancer models. Synthetic cannabinoids also demonstrate anti-proliferative effects on tumor progression. Despite their potential, cannabinoids’ psychoactive effects may hinder their advancement in cancer therapy. Non-psychoactive cannabinoids like cannabidiol, constituting up to 40% of cannabis extracts, exhibit pharmacological effects without causing undesirable psychoactive side effects, presenting a favorable risk–benefit profile. Cannabinoid agonists bind to canonical cannabinoid receptors 1 or cannabinoid receptors 2, modulating cancer-related pathways and inducing cell death. Additionally, cannabinoids can act through other receptors or can be receptor-independent, inhibiting pathways like PI3K-Akt and activating MAPK pathways, resulting in apoptotic death. Cannabinoids also induce the synthesis of ceramide, which activates an endoplasmic reticulum (ER) stress-related signaling pathway, leading to cell death by autophagy. Furthermore, cannabinoids exert anti-angiogenesis effects by blocking the vascular endothelial growth factor pathway and demonstrate anti-invasiveness and anti-metastasis actions. With abundant scientific literature supporting cannabinoids’ anti-cancer properties, there is a pressing need for more clinical studies to delve deeper into their potential. Several trials have already been initiated, focusing on diagnoses such as glioblastoma multiforme, where cannabinoids have shown promise (Figure 2).

For example, a study demonstrated that combining nabiximol spray with Temozolomide was well-tolerated by glioblastoma patients, leading to a notable difference in survival rates (83% after 1 year of nabiximol treatment compared to 44% in placebo-treated patients). Prior to this, an initial pilot trial explored the effects of intracranial Δ9-THC administration in patients with recurrent glioblastoma, uncovering a reduction in tumor proliferation in two out of the nine patients involved [38,39,40,41,42,43,46,47,48,49].

Another study investigated the efficacy and impact of cannabis use in oncology patients, comprising 68 individuals with metastatic disease beginning immunotherapy, among whom 34 were cannabis users. Cannabis consumption commenced between 9 months and 2 weeks prior to starting immunotherapy, with non-small cell lung cancer and melanoma being the predominant diagnoses. Notably, patients using cannabis demonstrated a median time to tumor progression of 3.4 months, in contrast to 13.1 months in non-users (*p* = 0.0025). Additionally, the median survival for cannabis users was 6.4 months, significantly shorter than the 28.5 months observed in non-users (*p* = 0.00094). This stark disparity in both disease progression and survival rates prompts significant inquiry. A noteworthy statistical distinction between the two groups in this non-randomized observational analysis was that 24% of cannabis users received immunotherapy as first-line therapy, compared to 46% of non-users (*p* = 0.03). The majority of cannabis users receiving immunotherapy as a second or third-line intervention could potentially contribute to some of the outcome differences. Furthermore, cannabis users experienced fewer immune-related adverse events, possibly due to the anti-inflammatory properties of cannabis, which could impact the efficacy of immunotherapy (Table 1) [44,45,50,51,52,53,54,55,56,57,58,59].

## 7. The Therapeutic Role of Cannabis in Managing Cancer Symptoms

### 7.1. Appetite Improvement

Cannabinoids are recognized for their efficacy as anti-emetic agents. Functionally, cannabinoid receptor 1, pivotal in this process, mitigates the emetic response by suppressing the release of excitatory neurotransmitters. Remarkably, cannabinoid receptor 1 is present on dopaminergic, noradrenergic, and other neurons located within brain regions that govern nausea and vomiting. Therefore, recently, the use of cannabis, particularly compounds like Δ9-THC and cannabidiol, has shown promise in stimulating appetite and managing symptoms in cancer patients. Studies have demonstrated that cannabis can increase caloric intake by 40%, and this increase was observed throughout the day, indicating a consistent effect of cannabis on appetite stimulation, although its effectiveness in promoting weight gain may vary. Interestingly, the increase in weight was primarily due to snacks, particularly sweet solid items. Studies examining the efficacy of dronabinol (at a dose of 2.5 mg), megestrol acetate (at a dose of 800 mg), or both have shown that among participants, megestrol exhibited the highest rate of appetite improvement, with 75% of patients experiencing increased appetite, followed by 66% for both compounds combined, and 49% for the dronabinol group. Additionally, megestrol was associated with a notable increase in weight gain, with 11% of patients exhibiting weight gain exceeding 10%, compared to only 3% for dronabinol [57,58,59,60,61,62]. Similarly, other cannabinoid medications like nabilone have shown mixed results in promoting weight gain in cancer patients. Recent studies investigating Δ9-THC and cannabidiol oil-based capsules have shown promising results in terms of appetite stimulation and improvements in mood, quality of life, and symptom management, although adverse effects have been reported. The combination of Δ9-THC and cannabidiol in cannabis preparations may influence appetite stimulation differently, with higher cannabidiol strains reported to produce less appetite stimulation and anxiety (Table 2) [59,62,63,64,65,66,67,68].

### 7.2. Pain Management

Patients with cancer frequently experience chronic pain and commonly resort to opioid analgesics for relief. The most common causes of pain include spinal cord compression or injury, chemotherapy, pathological fractures, bone metastasis, and metastases exerting pressure on the nerves. However, the use of opioids can pose significant risks, including drug dependence and incorrect dosing, especially when considering variations based on individual genetics or state regulations. Cannabinoids have long been recognized for their potential in pain management, with emerging research indicating their involvement in modulating nociceptive transmission [2,11,12,59,69,70,71,72,73,74,75,76,77,78,79]. Recent studies have underscored the widespread activity of the endocannabinoid system in pain regulation, particularly targeting the affective aspects of pain, attributed to the distribution of cannabinoid receptors in regions of the brain associated with emotions and cognition. It was reported that high levels of the cannabinoid 1 receptors are prominently found in brain areas regulating nociceptive processing. Although initially believed to modulate pain through similar pathways, opioids and cannabinoids exert their analgesic effects via distinct receptors. Notably, cannabinoids’ analgesic effects are unaffected by opioid antagonists. Moreover, both cannabinoid 1 receptors and cannabinoid 2 receptors agonists demonstrate peripheral and central analgesic actions, potentially aided by anti-inflammatory effects attributed to cannabinoids and terpenoids. The report emphasized that cannabis holds significant promise for pain relief, particularly in neuropathic pain conditions like HIV-related peripheral neuropathy. Encouragingly, a trial involving vaporized cannabis in diabetic neuropathy yielded positive results [2,11,12,59,69,70,71,72,73,74,75,76,77,78,79].

In the realm of cancer care, cannabinoids exhibit effectiveness in managing chemotherapy-induced peripheral neuropathy (CIPN) in animal models and some clinical settings. However, the evidence remains sparse, with only one published controlled trial assessing a cannabis-based medicine for CIPN. This trial, involving 16 patients randomized to nabiximols or placebo, showed no overall difference between groups. However, responder analysis revealed significant pain reduction in a subset of patients, with a mean pain reduction of 2.6 points on a 0–10 scale. The calculated number needed to treat for one patient to respond was five, suggesting potential efficacy. Notably, ongoing trials on cannabis-based medicines for CIPN aim to address this gap in evidence, highlighting a growing interest in this area. Observational studies offer supplementary insights, as seen in one of the analyses of cancer patients undergoing chemotherapy. Among those using cannabis, a lower incidence of grade 2–3 peripheral neuropathy was observed compared to non-users, suggesting a potential protective effect. Additionally, these studies suggest a possible role for cannabis in reducing opioid use among cancer patients for pain management. For instance, among patients diagnosed with colorectal cancer who commenced oxaliplatin and 5FU treatment, the level of protection was notably greater in those who began using cannabis before initiating oxaliplatin (75%) compared to those who started cannabis afterward (46.2%) (*p* < 0.001). Additionally, a study involving 2000 cancer patients using cannabis revealed that among the 344 individuals using opiates at baseline, 36% had ceased their opiate use, and 10% had reduced their dosage within 6 months. While data on cannabis-based medicines for non-neuropathic pain remain limited, trials investigating nabiximols in cancer patients have yielded mixed results. Meta-analyses of these trials indicated no significant difference in average pain scores between nabiximols and placebo, albeit with a higher risk of adverse events associated with cannabinoids. Overall, while randomized controlled trials pose challenges in evaluating cannabis as a Schedule 1 botanical, observational studies and ongoing clinical trials provide valuable insights into its potential therapeutic role in pain management, particularly in cancer care. Previous studies, as indicated in the text, have shown promising results regarding the efficacy of cannabinoids in managing pain, including chemotherapy-induced peripheral neuropathy, and their potential to reduce opioid use in cancer patients (Table 3) [2,11,12,59,69,70,71,72,73,74,75,76,77,78,79,80,81].

### 7.3. Nausea and Vomiting

Several academies of science reported that oral cannabinoids are effective antiemetics for adults experiencing chemotherapy-induced nausea and vomiting. In addition, a meta-analysis of these earlier studies investigating Δ9-THC pharmaceuticals like dronabinol and nabilone consistently showed their efficacy compared to placebo and standard antiemetics. However, more recent analyses, including a Cochrane review comprising 23 randomized controlled trials, have raised concerns about increased side effects and methodological limitations in these trials. Despite this evidence, the American Society of Clinical Oncology’s expert panel remains cautious, citing insufficient data to recommend medical marijuana for preventing nausea and vomiting in cancer patients undergoing chemotherapy or radiation therapy [2,11,82,83,84,85,86,87].

Clinical trials investigating cannabis-based medicines have shown promising results. For instance, a phase II trial of nabiximols demonstrated efficacy in reducing chemotherapy-induced nausea and vomiting, while a larger randomized trial of an oral Δ9-THC:CBD cannabis extract showed a significant improvement in complete response rates compared to placebo. This trial involved 81 cancer patients receiving emetogenic intravenous chemotherapy, with persistent nausea and vomiting despite standard antiemetics. Patients self-titrated with capsules containing CBD and Δ9-THC each at 2.5 mg three times daily or identical placebo capsules in a crossover design. They were then allowed to choose which they preferred for a third cycle. The complete response was improved from 14% to 25% with the Δ9-THC:CBD (RR 1.77; 1.12–2.79, *p* = 0.041). Despite self-reported moderate-to-severe adverse events being more frequent while receiving Δ9-THC:CBD (31%) compared to placebo (7%) (*p* = 0.002), 83% of the participants preferred cannabis to placebo. Evidence from medical practitioners’ observations, alongside patient-reported experiences, provides additional backing for the antiemetic effectiveness of cannabis. Patients across various clinical contexts have reported notable alleviation of nausea symptoms after consuming cannabis, especially products with higher Δ9-THC levels like flower and concentrates. Furthermore, data from 866 individuals provide additional affirmation of cannabis’ efficacy in treating nausea (Table 4) [80,81,82,83,84,85,86,87,88,89,90,91].

### 7.4. Insomnia

Issues with sleep onset and latency are widespread concerns among cancer patients and can affect nearly 19% of the overall population. Cannabis is commonly sought after by patients as a solution for insomnia. Some research suggests that short-term, high-dose CBD may assist in reducing sleep onset and prolonging sleep duration, possibly due to its anxiolytic properties. Conversely, conflicting evidence indicates that discontinuing cannabis after prolonged use may exacerbate or induce insomnia. Chronic pain often interferes with an individual’s ability to achieve restful sleep. Studies, primarily involving nabiximols, are starting to investigate how cannabinoids might address sleep disturbances in the context of pain. While many study participants subjectively reported enhanced sleep quality, this improvement may be more closely linked to reduced pain levels rather than alterations in sleep biology. Frequent use of cannabis, particularly high-Δ9-THC products, can lead to tolerance and may drive individuals to self-titrate, resulting in excessive Δ9-THC consumption over prolonged periods in an effort to enhance sleep. It is important to note that despite many patients turning to cannabis to alleviate sleep issues, evidence-based guidance on dosing or product composition is lacking. Given the longer half-life of oral or sublingual products, these formulations may be preferable for improving sleep duration (Table 5) [92,93,94,95,96].

## 8. Side Effects of Cannabis

Occasional cannabis use can elicit diverse psychoactive responses. While Δ9-THC commonly elicits feelings of euphoria, relaxation, and heightened sensory perception in most users, it can trigger anxiety and panic in others. Sensory distortions are also prevalent, and the intensity of psychomotor, cognitive, and behavioral disruptions tends to increase with dosage [2,16,58,59].

**Figure 3:**
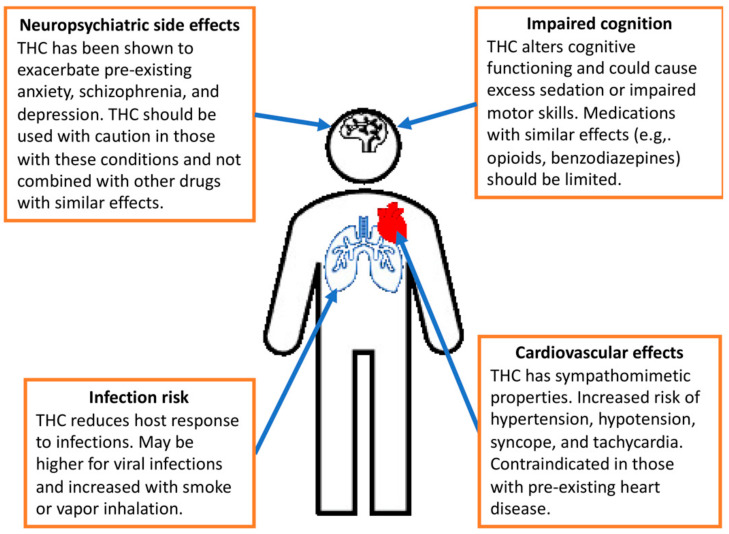
The primary adverse effects of tetrahydrocannabinol (THC) [99].

A comprehensive review examining over 3600 reports on synthetic cannabinoid toxicity identifies a range of physiological (nausea/vomiting and hypertension and tachycardia), emotional (agitation, irritability, paranoia), behavioral (drowsiness, aggression), and perceptual (hallucinations) symptoms. Tachycardia (30.2%), agitation (13.5%), drowsiness (12.3%), nausea/vomiting (8.2%), and hallucinations (7.6%) are among the most prevalent adverse effects, with fatalities or severe outcomes being rare (death 0.2%, stroke 0.1%, myocardial infarction 0.09%). In line with these findings, an analysis of more than 250 reports involving over 4000 cases and 26 deaths indicates predominantly mild to moderate presentations, typically in young males experiencing symptoms such as tachycardia (37–77%), agitation (16–41%), and nausea (13–94%), often requiring only symptomatic management and short hospital stays [2,16,58,59,92,93,94,95,96,97,98]. Unpleasant and occasionally severe symptoms associated with cannabis use include depersonalization, altered perception of time, paranoia, and anti-cholinergic effects (double vision, increased body temperature, dry mouth, urinary retention, anhidrosis, decreased heart rate). Δ9-THC may also induce orthostatic hypotension and reflex tachycardia lasting up to three hours after consumption, along with ataxia and reduced muscle strength. While sporadic marijuana use is generally deemed low-risk, withdrawal symptoms may include severe depressive episodes and suicidal thoughts. Additionally, reports suggest an increased risk of systolic hypertension, ischemic stroke, and ventricular arrhythmias with chronic marijuana consumption. However, prolonged use may result in tolerance to its cardiovascular effects, potentially through receptor downregulation or reduced susceptibility [92,93,94,95,96,97,98], Figure 3 [99].

## 9. Discussion

Cancer stands as the second-leading cause of death in the United States overall and the primary cause among individuals under 85 years old. By the year 2024, it is projected that over 2 million new cancer cases and more than 600,000 cancer-related deaths will occur in the United States alone. Encouragingly, cancer mortality has shown a decline up to 2021, preventing over 4 million deaths since 1991 due to factors such as decreased smoking rates, advancements in early detection methods for certain cancers, and improved treatment options across both adjuvant and metastatic settings. The cancer is characterized by the rapid proliferation of abnormal cells exceeding their usual boundaries, cancer manifests as a complex disease process. Tumor development progresses through multiple stages initiated by DNA damage, leading to mutations, disruptions in the cell cycle, and suppression of apoptosis [11,100,101,102,103,104]. Given the widespread presence of the cannabinoid system throughout the body, significant attention has been directed towards exploring the role of cannabinoids in cancer research in recent years. In the field of oncology, cannabinoids find application primarily in alleviating therapy- and tumor-related symptoms, with research in this area dating back to the early 1970s. Presently, as many as 70% of oncologists report having discussions with their patients regarding the use of cannabis products. However, they also acknowledge a lack of comprehensive information to offer robust recommendations [100,101,102,103,104].

This article provides a comprehensive overview of the therapeutic potential of cannabis in cancer management, focusing on its anti-tumor effects, symptom management, and associated side effects. Phytocannabinoids, naturally occurring compounds found in various plants including cannabis, exert their effects through the endogenous cannabinoid system. This system involves cannabinoid receptors, particularly cannabinoid receptor 1 and cannabinoid receptor 2, which are integral in regulating physiological functions such as mood, appetite, and pain perception. Understanding the mechanisms of cannabinoid receptor signaling is crucial for elucidating the therapeutic potential of cannabis-based medicines [20,21,22,23,24,25,26,27,28].

A key strength of this article lies in its thorough coverage of different aspects of cannabis, including its pharmacology, physiology, history, as well as clinical use profile, making it a valuable resource for both clinicians and patients seeking to understand cannabis’ role in oncology. Additionally, the article successfully highlights the knowledge gaps in this area, emphasizing the areas of need for more studies to validate the therapeutic efficacy and safety of cannabis in cancer management. The main limitation of the article is the potential bias resulting from the limited availability of high-quality clinical data and studies on the subject as well as the narrow scope of existing studies.

Cannabis-derived compounds, particularly Δ9-THC and cannabidiol, have demonstrated significant anti-tumor actions in preclinical studies and some clinical settings. These compounds inhibit tumor proliferation, induce apoptosis, and interfere with processes like angiogenesis and metastasis. Despite promising findings, the psychoactive effects of cannabinoids pose challenges in their clinical application. Non-psychoactive cannabinoids like cannabidiol offer a favorable risk–benefit profile, potentially circumventing concerns associated with psychoactivity [38,39,40,41,42,43,46,47,48,49].

Cannabis shows promise in managing various cancer-related symptoms, including pain, nausea, vomiting, and appetite loss. Cannabinoids interact with neurotransmitter systems involved in pain perception and emesis, offering an alternative or adjunctive therapy to conventional treatments. However, the efficacy of cannabis-based medicines in symptom management warrants further investigation through well-designed clinical trials to establish safety and efficacy profiles [2,3,4,7,8,9,10,11].

While cannabis offers therapeutic benefits, it is not without side effects. Acute effects of cannabis use include psychoactive responses such as euphoria, anxiety, and sensory distortions. Synthetic cannabinoids, in particular, have been associated with adverse physiological, emotional, and perceptual symptoms, underscoring the importance of caution in their consumption. Long-term cannabis use may also lead to tolerance, withdrawal symptoms, and cardiovascular risks, highlighting the need for informed decision-making and monitoring of cannabis use, especially in vulnerable populations [97,98,99,100,101].

Despite the interest and effectiveness of cannabis for cancer patients, and as is known, several ongoing research endeavors focusing on the mechanisms of cannabinoid action, novel delivery systems, and personalized approaches to cannabinoid therapy hold promise in optimizing cancer care. The information that is presented in this article underscores the need for further clinical studies to validate the therapeutic efficacy of cannabis in cancer management. Randomized controlled trials assessing the safety and efficacy of cannabis-based medicines in symptom control, disease progression, and quality of life are essential for guiding clinical practice and regulatory decision-making.

## 10. Conclusions

While cannabis holds promise in cancer therapy and symptom management, its clinical use demands meticulous evaluation of both its therapeutic benefits and potential risks. Effective utilization of cannabis in oncology necessitates collaborative endeavors among researchers, clinicians, and regulatory bodies to deepen our comprehension of its pharmacology and optimize its therapeutic efficacy. These findings underscore the imperative for continued research to solidify cannabis-based medicines as viable options for managing pain, nausea, vomiting, and other symptoms in oncological practice.

## Figures and Tables

**Table 1:** Summary table of studies exploring anti-tumor effects of cannabis.

Authors	Purpose	Design	Key Findings
Kaur R, Javid FA [16]	Evaluate the potential of cannabinoids in ovarian cancer treatment	Review article	Cannabinoids’ potential in treating gynecological cancers, focusing on non-psychoactive options like CBD, it’s anti-tumor properties and interactions with estrogen.
Davis MP [38]	Analyze the role of cannabinoids in symptom management and cancer therapy	Review article	Cannabinoids’ effects on cancer pain, nausea, appetite, and tumor progression, comparing their efficacy to other treatments.
Munson AE et al. [39]	Investigate the anti-neoplastic activity of cannabinoids	Experimental study	Δ9-THC, Δ8-THC, and CBN effects on Lewis lung adenocarcinoma growth, showing tumor retardation and increased survival, compared to the ineffectiveness of CBD in tumor inhibition.
Velasco G, Sanchez C, Guzman M [40]	Explore the use of cannabinoids as anti-tumor agents	Review article	Molecular mechanisms of cannabinoids’ anti-tumor action in various cancers, including emerging resistance mechanisms and opportunities for combination therapy approaches.
Pisanti S et al. [41]	Discuss the endocannabinoid system in cancer	Review article	Endocannabinoid system’s anti-tumor effects, role in cancer pathogenesis, alternative view of cannabinoid receptors as tumor promoters, emerging crosstalk with other receptors, and involvement in lipid pathways, including MAGL’s role in tumor metabolism.
Mimeault M et al. [42]	Study the effects of anandamide in prostate cancer	Experimental study	Anandamide anti-proliferative effects in prostatic cancer cell lines through downregulation of EGFR and ceramide production.
Caffarel MM et al. [43]	Investigate the role of cannabinoids in breast cancer	Experimental study	Δ9-THC and JWH-133 reduce tumor growth, metastases, and angiogenesis in mouse models by suppressing cancer cell proliferation, triggering apoptosis, and partially inhibiting the pro-tumorigenic Akt pathway, with ErbB2-positive tumors expressing the CB2 receptor.
De Petrocellis L, Di Marzo V [44]	Introduce the endocannabinoid system	Review article	Overview of the endocannabinoid system’s role in various physiological processes.
Mechoulam R et al. [45]	Discuss advances in cannabidiol research	Review article	Cannabidiol as a therapeutic and pharmacological agent with potential benefits and no psychoactive effects.
Pantoja-Ruiz C et al. [46]	Review the relationship between cannabis and pain management	Review article	Cannabis in the management of different pain etiologies including cancer pain.
Pagano C et al. [47]	Investigate the molecular mechanisms of cannabinoids in cancer progression	Review article	Cannabinoids interfere with intracellular cancer progression pathways such as cell cycle, apoptosis, migration, invasion, and angiogenesis.
Hinz B, Ramer R [48]	Assess cannabinoids as anti-cancer drugs	Review of article	Cannabinoids show significant potential in preclinical cancer models, demonstrating anti-tumor activity.
Javid FA et al. [49]	Explore cannabinoid pharmacology in cancer research	Review article	Cannabinoids hold promise as new therapeutic agents for cancer, with potential for novel treatment strategies.
Ghasemiesfe M et al. [50]	Assess marijuana use and cancer risk	Systematic review and meta-analysis	Evidence of long-term marijuana use leads to an increased risk of testicular germ cell tumors. Examines evidence for associations with lung, head and neck, oral, urogenital, and other cancers. Further research is needed to clarify these potential relationships.
Chusid MJ et al. [51]	Examine pulmonary risks from marijuana smoke	Case report	Identified pulmonary aspergillosis from contaminated marijuana in immunocompromised individuals.
Wallace JM et al. [52]	Identify risk factors for Aspergillus in HIV patients	Case-control study	Found associations between Aspergillus in respiratory specimens and HIV, evaluating the significance of marijuana us as a risk factor.
Kocis PT, Vrana KE [53]	Explore cannabinoids interaction with CYP enzymes	Review article	Cannabinoids can serve as both inhibitors and inducers of various CYP enzymes UDP-glucuronosyltransferase enzymes, which could lead to significant interactions with medications metabolized by these enzymes.
Engels FK et al. [54]	Study medicinal cannabis and chemotherapy drug interaction	Clinical drug-interaction study	The study involving 24 cancer patients found that medicinal cannabis, administered as herbal tea, did not significantly affect the CYP3A-mediated pharmacokinetics of irinotecan or docetaxel.
Yamaori S et al. [55]	Characterize cannabinoids interaction with CYP1 enzymes	Biochemical study	CBD and CBN are potent, isoform-selective inhibitors of human CYP1 enzymes, particularly CYP1A1, with CBD acting as a mechanism-based inhibitor, affecting drug interactions and the bioactivation of procarcinogens.
Taha T et al. [56]	Investigate cannabis impact on tumor response to nivolumab	Retrospective observational study	The study included 140 patients with advanced melanoma, non-small cell lung cancer, and renal cell carcinoma; 89 received nivolumab alone, and 51 combined it with cannabis. Cannabis use significantly lowered the tumor response rate (15.9% vs. 37.5%) but did not significantly affect progression-free or overall survival.
Bar-Sela G et al. [57]	Correlate cannabis use with immunotherapy outcomes	Prospective observational cohort study	The study involving 102 advanced cancer patients (34 cannabis users) found that cannabis consumption during immunotherapy was associated with significantly worse clinical outcomes, including shorter time to tumor progression (3.4 vs. 13.1 months) and overall survival (6.4 vs. 28.5 months), along with lower lymphocyte counts and fewer immune-related adverse events, suggesting a potential immunosuppressive effect.
Abrams DI, Guzmán M [58]	Discuss potential of cannabis in cancer treatment	Commentary	Explored whether cannabis could be effective in curing cancer and the basis for this opinion
Abrams DI [59]	Review cannabis-based medicines in cancer care	Review article	This review compares results from various studies and clinical trials on managing cancer symptoms and explores cannabinoids’ potential anti-cancer effects, including their use in treating glioblastoma multiforme.

**Table 2:** Summary table of studies exploring cannabis as appetite stimulant.

Authors	Purpose	Research Design	Key Findings
Jatoi A et al. [62]	Compare dronabinol, megestrol acetate, and combination therapy for cancer-associated anorexia	Randomized clinical trial	The study included 469 advanced cancer patients; 157 received megestrol acetate, 157 received dronabinol, and 155 received both. Megestrol acetate improved appetite (75% vs. 49%) and weight gain (11% vs. 3%) compared to dronabinol, with no additional benefit from combination therapy.
Turcott JG et al. [63]	Evaluate nabilone’s effects on appetite and quality of life in lung cancer patients	Randomized, double-blind clinical study	The study included 47 patients with advanced non-small cell lung cancer; 24 received Nabilone and 23 received a placebo. Nabilone significantly increased caloric intake (342 kcal vs. placebo) and carbohydrate intake (64 g vs. placebo), and improved quality of life, particularly in role, emotional, and social functioning, as well as pain and insomnia.
Bar-Sela G et al. [64]	Assess cannabis capsules’ effects on cancer-related cachexia and anorexia syndrome	Pilot study	The study included 17 advanced cancer patients who received dosage-controlled cannabis capsules; 3 patients (17.6%) experienced a ≥10% weight gain, with improvements in appetite and quality of life, while some reported mild side effects.
PDQ Supportive and Palliative Care Editorial Board [65]	Review nausea and vomiting management related to cancer treatment	Health information summary	Highlights the role of cannabinoids in managing cancer-related nausea and vomiting, with implications for appetite improvement.
Sharkey, K.A.; Wiley, J.W. [66]	Explore the role of the endocannabinoid system in the brain–gut axis	Review article	Discusses how the endocannabinoid system influences appetite and gut function, relevant to cancer-related cachexia.
Sharkey, K.A.; Darmani, N.A.; Parker, L.A. [67]	Study the regulation of nausea and vomiting by cannabinoids	Review article	Highlights the role of cannabinoids in reducing nausea, with potential effects on appetite stimulation.
Limebeer, C.L.; Rock, E.M.; Mechoulam, R.; Parker, L.A. [68]	Investigate the anti-nausea effects of CB1 agonists	Experimental study	Nausea-relieving effects of CB1 agonists in rats are mediated by their action in the visceral insular cortex. The central administration of the CB1 agonist suppressed LiCl-induced nausea, an effect blocked by a CB1 antagonist.
Abrams DI [59]	Review the use of cannabis and cannabinoids in cancer care	Review article	Cannabis and cannabinoids are effective in improving appetite and managing cachexia in cancer patients.

**Table 3:** Summary table of studies exploring cannabis in pain management.

Authors	Purpose	Research Design	Key Findings
Dzierżanowski T [2]	Review the prospects of cannabinoids in oncology and palliative care	Review article	Cannabinoids show potential in managing pain in oncology and palliative care settings.
Pagano C et al. [9]	Discuss the therapeutic use of cannabinoids in clinical practice	Review article	Cannabinoids are effective in pain relief and show therapeutic potential in clinical settings.
Buchtova T et al. [10]	Study drug–drug interactions of cannabidiol with chemotherapy drugs	Review article	Cannabidiol interacts with standard chemotherapeutics but also offers pain management benefits.
Abrams DI [54]	Review the use of cannabis and cannabinoids in cancer care	Review article	Cannabinoids are effective in managing cancer-related pain, including neuropathic pain.
LiverTox [64]	Discuss nabilone’s role in drug-induced liver injury	Health information summary	Nabilone is used for pain management focusing on safety and liver health.
Bar-Lev Schleider L et al. [65]	Analyze the safety and efficacy of medical cannabis in cancer patients	Prospective analysis	The study included 2970 cancer patients. Medical cannabis improved symptoms in 95.9% of patients, addressing issues like pain (77.7%), sleep problems (78.4%), and nausea (64.6%), and was found to be a well-tolerated and safe option for palliative care.
Vučković S et al. [66]	Explore cannabinoids and their role in pain management	Review article	Cannabinoids offer significant pain relief, with new insights from old molecules.
Abrams DI et al. [67]	Test cannabis for painful HIV-associated sensory neuropathy	Randomized placebo-controlled study	50 adults with HIV-associated sensory neuropathy found that smoked cannabis significantly reduced chronic neuropathic pain by 34% compared to 17% with placebo, with 52% of the cannabis group achieving over 30% pain reduction.
Andreae MH et al. [68]	Meta-analyze inhaled cannabis for chronic neuropathic pain	Meta-analysis	Inhaled cannabis provided significant relief for chronic neuropathic pain.
Wallace MS et al. [69]	Evaluate the efficacy of inhaled cannabis on diabetic neuropathy	Randomized, double-blinded, placebo-controlled crossover study	16 patients with painful diabetic peripheral neuropathy were exposed to placebo and three doses of inhaled cannabis (1%, 4%, and 7% THC) in a crossover design, showing a dose-dependent reduction in pain, with significant pain relief at higher doses but also cognitive impairment at the highest dose.
Rahn EJ, Makriyannis A, Hohmann AG [70]	Study the effects of CB1 and CB2 activation on neuropathic pain	Experimental study	Cannabinoids suppress vincristine-induced mechanical allodynia in rats through activation of CB1 and CB2 receptors, with the anti-allodynic effects primarily mediated at the spinal cord level.
Khasabova IA et al. [71]	Investigate CB1 receptor’s role in reducing chemotherapy-induced pain and neurotoxicity	Experimental study	Enhancing anandamide signaling through the CB1 receptor, using the inhibitor URB597, effectively reduces cisplatin-induced mechanical and heat hyperalgesia in mice.
Ward SJ et al. [72]	Examine cannabidiol’s effects on paclitaxel-induced neuropathic pain	Experimental study	Cannabidiol prevents paclitaxel-induced neuropathic pain in mice through the 5-HT(1A) receptor system without causing cognitive impairment, conditioned rewarding effects, or reducing the chemotherapy’s effectiveness against breast cancer cells.
Lynch ME, Cesar-Rittenberg P, Hohmann AG [73]	Evaluate oral mucosal cannabinoid extract for chemotherapy-induced neuropathic pain	Double-blind, placebo-controlled trial	16 patients with chemotherapy-induced neuropathic pain; participants received nabiximols or placebo. While there was no significant difference in overall pain reduction between groups, five participants reported a meaningful pain decrease.
Waissengrin B et al. [74]	Analyze the effect of cannabis on oxaliplatin-induced peripheral neuropathy	Retrospective analysis	513 patients were treated with oxaliplatin; 248 received cannabis and 265 served as controls. Cannabis significantly reduced the rate of chemotherapy-induced peripheral neuropathy (15.3% vs. 27.9%), with a more pronounced protective effect when cannabis was used before oxaliplatin treatment.
Boland EG et al. [75]	Systematically review cannabinoids for adult cancer-related pain	Systematic review and meta-analysis	Cannabinoids were effective in managing cancer-related pain.
Abrams DI [76]	Explore cannabinoid-opioid interactions in chronic pain	Clinical pharmacology study	The study included 21 chronic pain patients on morphine or oxycodone and found that adding vaporized cannabis significantly reduced pain by 27% without altering plasma opioid levels, suggesting that cannabis may enhance opioid analgesia and potentially allow for lower opioid doses with a reduction in side effects.

**Table 4:** Summary table of studies exploring cannabis in management of nausea and vomiting.

Authors	Purpose	Research Design	Key Findings
Dzierżanowski T [2]	Review the prospects of cannabinoids in oncology and palliative care	Review article	Cannabinoids show potential in managing nausea and vomiting in oncology settings.
Pagano C et al. [11]	Discuss therapeutic use of cannabinoids in clinical practice	Review article	Cannabinoids are effective in managing nausea and vomiting, especially in chemotherapy patients.
Tramèr MR et al. [82]	Quantitative systematic review of cannabinoids for chemotherapy-induced nausea and vomiting	Review article	Cannabinoids were found to be effective in controlling chemotherapy-induced nausea and vomiting.
Ben Amar M [83]	Review cannabinoids’ therapeutic potential	Review article	Cannabinoids are effective in treating nausea and vomiting, with particular efficacy in chemotherapy-induced cases.
Smith LA et al. [84]	Review cannabinoids for nausea and vomiting in chemotherapy patients	Systematic review	Cannabinoids are effective for nausea and vomiting management in adults receiving chemotherapy.
Tafelski S et al. [85]	Review on cannabinoids for chemotherapy-induced nausea and vomiting	Systematic review	Cannabinoids are effective and generally well-tolerated for managing chemotherapy-induced nausea and vomiting.
Schussel V et al. [86]	Overview of systematic reviews on cannabinoids for chemotherapy-related nausea and vomiting	Review article	Supported efficacy of cannabinoids in managing chemotherapy-induced nausea and vomiting.
Chow R et al. [87]	Systematic review and meta-analysis of oral cannabinoids for nausea and vomiting	Systematic and meta-analysis	Oral cannabinoids are effective in preventing chemotherapy-induced nausea and vomiting.
Hesketh PJ et al. [88]	Update on antiemetic guidelines by ASCO	Clinical guideline	Cannabinoids are included as effective options for managing chemotherapy-induced nausea and vomiting.
Duran M et al. [89]	Evaluate the efficacy and safety of or mucosal cannabis extract for chemotherapy-induced nausea and vomiting	Phase II clinical trial	16 patients with chemotherapy-induced nausea and vomiting; 7 were randomized to the cannabis-based medicine group and 9 to the placebo group. Those in the CBM group had a higher complete response rate (71.4% vs. 22.2%) compared to placebo, with CBM being well tolerated despite a higher incidence of adverse events (86% vs. 67%).
Grimison P et al. [90]	Randomized placebo-controlled trial of THC–CBD extract for refractory chemotherapy-induced nausea and vomiting	Phase II crossover trial	81 participants with refractory chemotherapy-induced nausea and vomiting; those treated with oral THC–CBD had an improved complete response rate (25% vs. 14%) compared to placebo, with 31% experiencing moderate or severe side effects, and 83% preferring THC–CBD over placebo.
Zikos TA et al. [91]	Assess the perceived effectiveness of marijuana, ondansetron, and promethazine for gastrointestinal nausea	Survey	Survey of 153 patients with chronic gastrointestinal nausea found that marijuana, ondansetron, and promethazine were rated significantly more effective than other treatments. Marijuana was particularly beneficial for severe nausea.

**Table 5:** Summary table of studies exploring cannabis in management of insomnia.

Authors	Purpose	Research Design	Key Findings
Davidson JR et al. [92]	Explore sleep disturbances in cancer patients	Survey	Sleep disturbances are common in cancer patients.
Carlini EA, Cunha JM, Paulo S [93]	Investigate the hypnotic and anti-epileptic effects of cannabidiol	Clinical review study	Cannabidiol was safe and at various doses, improved sleep, and reduced seizures in epilepsy patients, with no significant psychotropic or toxic effects observed.
Betthauser K et al. [94]	Assess the use of cannabinoids in veterans with PTSD	Survey	Cannabinoids, including cannabis, were effective in managing sleep disturbances in veterans with PTSD, relevant to insomnia management.
Babson KA, Bonn-Miller MO [95]	Review sleep disturbances and cannabis use	Review article	Cannabis use was associated with alleviating sleep disturbances, but cessation could lead to withdrawal-related insomnia.
Sznitman SR et al. [96]	Study medical cannabis use and insomnia in older adults with chronic pain	Cross-sectional study	128 chronic pain patients over age 50; 66 were medical cannabis users and 62 were non-users. Medical cannabis use was associated with fewer problems waking up at night, but frequent use correlated with increased sleep disturbances, suggesting potential tolerance to its sleep-inducing effects.

## Data Availability

The data presented in this study are available on request from the corresponding author.

## References

[1] Hoch E., Projektgruppe C.P.R., Friemel C., Schneider M., Pogarell O., Hasan A., Preuss U.W. (2019). Wirksamkeit und Sicherheit von Cannabisarzneimitteln: Ergebnisse der CaPRis-Studie. Bundesgesundheitsbl.

[2] Dzierżanowski T. (2019). Prospects for the Use of Cannabinoids in Oncology and Palliative Care Practice: A Review of the Evidence. Cancers.

[3] Whiting P.F., Wolff R.F., Deshpande S., Di Nisio M., Duffy S., Hernandez A.V., Keurentjes J.C., Lang S., Misso K., Ryder S. (2015). Cannabinoids for Medical Use: A Systematic Review and Meta-analysis. JAMA.

[4] Goyal H., Singla U., Gupta U., May E. (2017). Role of cannabis in digestive disorders. Eur. J. Gastroenterol. Hepatol..

[5] Braile M., Marcella S., Marone G., Galdiero M.R., Varricchi G., Loffredo S. (2021). The Interplay between the Immune and the Endocannabinoid Systems in Cancer. Cells.

[6] Braile M., Cristinziano L., Marcella S., Varricchi G., Marone G., Modestino L., Ferrara A.L., De Ciuceis A., Scala S., Galdiero M.R. (2021). LPS-mediated neutrophil VEGF-A release is modulated by cannabinoid receptor activation. J. Leukoc. Biol..

[7] Maccarrone M. (2020). Missing Pieces to the Endocannabinoid Puzzle. Trends Mol. Med..

[8] Matsuda L.A., Lolait S.J., Brownstein M.J., Young A.C., Bonner T.I. (1990). Structure of a cannabinoid receptor and functional expression of the cloned cDNA. Nature.

[9] Zou S., Kumar U. (2018). Cannabinoid Receptors and the Endocannabinoid System: Signaling and Function in the Central Nervous System. Int. J. Mol. Sci..

[10] Munro S., Thomas K.L., Abu-Shaar M. (1993). Molecular characterization of a peripheral receptor for cannabinoids. Nature.

[11] Pagano C., Navarra G., Coppola L., Avilia G., Bifulco M., Laezza C. (2022). Cannabinoids: Therapeutic Use in Clinical Practice. Int. J. Mol. Sciences..

[12] Buchtova T., Lukac D., Skrott Z., Chroma K., Bartek J., Mistrik M. (2023). Drug–Drug Interactions of Cannabidiol with Standard-of-Care Chemotherapeutics. Int. J. Mol. Sci..

[13] Scripture C.D., Figg W.D. (2006). Drug Interactions in Cancer Therapy. Nat. Rev. Cancer.

[14] Pergam S.A., Woodfield M.C., Lee C.M., Cheng G.-S., Baker K.K., Marquis S.R., Fann J.R. (2017). Cannabis Use among Patients at a Comprehensive Cancer Center in a State with Legalized Medicinal and Recreational Use. Cancer.

[15] Weiss M.C., Hibbs J.E., Buckley M.E., Danese S.R., Leitenberger A., Bollmann-Jenkins M., Meske S.W., Aliano-Ruiz K.E., McHugh T.W., Larson S.L. (2022). A Coala-T-Cannabis Survey Study of Breast Cancer Patients’ Use of Cannabis before, during, and after Treatment. Cancer.

[16] Kaur R., Javid F.A. (2023). Could cannabinoids provide a new hope for ovarian cancer patients?. Pharmacol. Res. Perspect..

[17] Touw M. (1981). The religious and medicinal uses of cannabis in China, India and Tibet. J. Psychoact. Drugs.

[18] Robinson S.M., Adinoff B. (2016). The classification of substance use disorders: Historical, contextual, and conceptual considerations. Behav. Sci..

[19] Zuardi A.W. (2006). History of cannabis as a medicine: A review. Rev. Bras. Psiquiatr..

[20] Chapman E.J., Edwards Z., Boland J.W., Maddocks M., Fettes L., Malia C., Mulvey M.R., I Bennett M. (2020). Practice review: Evidence-based and effective management of pain in patients with advanced cancer. Palliat. Med..

[21] Hanuš L.O., Meyer S.M., Muñoz E., Taglialatela-Scafati O., Appendino G. (2016). Phytocannabinoids: A unified critical inventory. Nat. Prod. Rep..

[22] Bonini S.A., Premoli M., Tambaro S., Kumar A., Maccarinelli G., Memo M., Mastinu A. (2018). Cannabis sativa: A comprehensive ethnopharmacological review of a medicinal plant with a long history. J. Ethnopharmacol..

[23] Milay L., Berman P., Shapira A., Guberman O., Meiri D. (2020). Metabolic Profiling of Cannabis Secondary Metabolites for Evaluation of Optimal Postharvest Storage Conditions. Front. Plant Sci..

[24] Hanuš L.O., Hod Y. (2020). Terpenes/Terpenoids in Cannabis: Are They Important?. Med. Cannabis Cannabinoids.

[25] Das P.C., Vista A.R., Tabil L.G., Baik O.-D. (2022). Postharvest operations of cannabis and their effect on cannabinoid content: A review. Bioengineering.

[26] Zhou H., Peng X., Hou T., Zhao N., Qiu M., Zhang X., Liang X. (2020). Identification of novel phytocannabinoids from Ganoderma by label-free dynamic mass redistribution assay. J. Ethnopharmacol..

[27] Castaneto M.S., Gorelick D.A., Desrosiers N.A., Hartman R.L., Pirard S., Huestis M.A. (2014). Synthetic cannabinoids: Epidemiology, pharmacodynamics, and clinical implications. Drug Alcohol. Depend..

[28] Koltai H., Shalev N. (2022). Anti-Cancer Activity of Cannabis sativa Phytocannabinoids: Molecular Mechanisms and Potential in the Fight against Ovarian Cancer and Stem Cells. Cancers.

[29] Keyhani S., Steigerwald S., Ishida J., Vali M., Cerdá M., Hasin D., Dollinger C., Yoo S.R., Cohen B.E. (2018). Risks and benefits of marijuana use a national survey of U.S. Adults. Ann. Intern. Med..

[30] Ghasemiesfe M., Ravi D., Vali M., Korenstein D., Arjomandi M., Frank J., Austin P.C., Keyhani S. (2018). Marijuana use, respiratory symptoms, and pulmonary function: A systematic review and meta-analysis. Ann. Intern. Med..

[31] Ravi D., Ghasemiesfe M., Korenstein D., Cascino T., Keyhani S. (2018). Associations between marijuana use and cardiovascular risk factors and outcomes a systematic review. Ann. Intern. Med..

[32] Spindle T.R., Cone E.J., Schlienz N.J., Mitchell J.M., Bigelow G.E., Flegel R., Hayes E., Vandrey R. (2018). Acute effects of smoked and vaporized cannabis in healthy adults who infrequently use cannabis: A crossover trial. JAMA Netw. Open.

[33] Worster B., Hajjar E.R., Handley N. (2022). Cannabis Use in Patients With Cancer: A Clinical Review. JCO Oncol. Pract..

[34] (2021). PR Newswire: The Worldwide Cannabis Food and Beverage Industry Is Expected to Grow at a CAGR of 15.15% between 2021 to 2026. https://www.prnewswire.com/news-releases/the-worldwide-cannabis-food-and-beverage-industry-is-expected-to-grow-at-a-cagr-of-15-15-between-2021-to-2026--301331375.html.

[35] Romero-Sandoval E.A., Kolano A.L., Alvarado-Vázquez P.A. (2017). Cannabis and cannabinoids for chronic pain. Curr. Rheumatol. Rep..

[36] Steigerwald S., Wong P.O., Khorasani A., Keyhani S. (2018). The form and content of cannabis products in the United States. J. Gen. Intern. Med..

[37] Grotenhermen F. (2003). Pharmacokinetics and pharmacodynamics of cannabinoids. Clin. Pharmacokinet..

[38] Davis M.P. (2016). Cannabinoids for symptom management and cancer therapy: The evidence. J. Natl. Compr. Cancer Netw..

[39] Munson A.E., Harris L.S., Friedman M.A., Dewey W.L., Carchman R.A. (1975). Antineoplastic activity of cannabinoids. J. Natl. Cancer Inst..

[40] Velasco G., Sanchez C., Guzman M. (2012). Towards the use of cannabinoids as antitumour agents. Nat. Rev. Cancer..

[41] Pisanti S., Picardi P., D'Alessandro A., Laezza C., Bifulco M. (2013). The endocannabinoid signaling system in cancer. Trends Pharmacol. Sci..

[42] Mimeault M., Pommery N., Wattez N., Bailly C., Henichart J.P. (2003). Anti-proliferative and apoptotic effects of anandamide in human prostatic cancer cell lines: Implication of epidermal growth factor receptor down-regulation and ceramide production. Prostate.

[43] Caffarel M.M., Andradas C., Mira E., Pérez-Gómez E., Cerutti C., Moreno-Bueno G., Flores J.M., García-Real I., Palacios J., Mañes S. (2010). Cannabinoids reduce ErbB2-driven breast cancer progression through Akt inhibition. Mol. Cancer..

[44] De Petrocellis L., Di Marzo V. (2009). An introduction to the endocannabinoid system: From the early to the latest concepts. Best. Pract. Res. Clin. Endocrinol. Metab..

[45] Mechoulam R., Peters M., Murillo-Rodriguez E., Hanus L.O. (2007). Cannabidiol—Recent advances. Chem. Biodivers..

[46] Pantoja-Ruiz C., Restrepo-Jimenez P., Castañeda-Cardona C., Ferreirós A., Rosselli D. (2022). Cannabis and pain: A scoping review. Braz. J. Anesthesiol..

[47] Pagano C., Navarra G., Coppola L., Bifulco M., Laezza C. (2021). Molecular mechanism of cannabinoids in cancer progression. Int. J. Mol. Sci..

[48] Hinz B., Ramer R. (2022). Cannabinoids as anticancer drugs: Current status of preclinical research. Br. J. Cancer..

[49] Javid F.A., Phillips R.M., Afshinjavid S., Verde R., Ligresti A. (2016). Cannabinoid pharmacology in cancer research: A new hope for cancer patients?. Eur. J. Pharmacol..

[50] Ghasemiesfe M., Barrow B., Leonard S., Keyhani S., Korenstein D. (2019). Association between marijuana use and risk of cancer: A systematic review and meta-analysis. JAMA Netw. Open.

[51] Chusid M.J., Gelfand J.A., Nutter C., Fauci A.S. (1975). Letter: Pulmonary aspergillosis, inhalation of contaminated marijuana smoke, chronic granulomatous disease. Ann. Intern. Med..

[52] Wallace J.M., Lim R., Browdy B.L., Hopewell P.C., Glassroth J., Rosen M.J., Reichman L.B., Kvale P.A. (1998). Risk factors and outcomes associated with identification of Aspergillus in respiratory specimens from persons with HIV disease. Pulmonary Complications of HIV Infection Study Group. Chest.

[53] Kocis P.T., Vrana K.E. (2020). Delta-9-tetrahydrocannabinol and cannabidiol drug-drug interactions. Med. Cannabis Cannabinoids.

[54] Engels F.K., de Jong F.A., Sparreboom A., Mathot R.A.A., Loos W.J., Kitzen J.J.E.M., de Bruijn P., Verweij J., Mathijssen R.H.J. (2007). Medicinal cannabis does not influence the clinical pharmacokinetics of irinotecan and docetaxel. Oncol..

[55] Yamaori S., Kushihara M., Yamamoto I., Watanabe K. (2010). Characterization of major phytocannabinoids, cannabidiol and cannabinol, as isoform-selective and potent inhibitors of human CYP1 enzymes. Biochem. Pharmacol..

[56] Taha T., Meiri D., Talhamy S., Wollner M., Peer A., Bar-Sela G. (2019). Cannabis impacts tumor response rate to nivolumab in patients with advanced malignancies. Oncologist.

[57] Bar-Sela G., Cohen I., Campisi-Pinto S., Lewitus G.M., Oz-Ari L., Jehassi A., Peer A., Turgeman I., Vernicova O., Berman P. (2020). Cannabis consumption used by cancer patients during immunotherapy correlates with poor clinical outcome. Cancers.

[58] Abrams D.I., Guzmán M. (2020). Can cannabis cure cancer?. JAMA Oncol..

[59] Abrams D.I. (2022). Cannabis, Cannabinoids and Cannabis-Based Medicines in Cancer Care. Integr. Cancer Ther..

[60] Foltin R.W., Fischman M.W., Byrne M.F. (1988). Effects of smoked marijuana on food intake and body weight of humans living in a residential laboratory. Appetite.

[61] Beal J.E., Olson R., Laubenstein L., Morales J.O., Bellman P., Yangco B., Lefkowitz L., Plasse T.F., Shepard K.V. (1995). Dronabinol as a treatment for anorexia associated with weight loss in patients with AIDS. J. Pain. Symptom Manag..

[62] Jatoi A., Windschitl H.E., Loprinzi C.L., Sloan J.A., Dakhil S.R., Mailliard J.A., Pundaleeka S., Kardinal C.G., Fitch T.R., Krook J.E. (2002). Dronabinol versus megestrol acetate versus combination therapy for cancer-associated anorexia: A North Central Cancer Treatment Group study. J. Clin. Oncol..

[63] Turcott J.G., Núñez M.d.R.G., Flores-Estrada D., Oñate-Ocaña L.F., Zatarain-Barrón Z.L., Barrón F., Arrieta O. (2018). The effect of nabilone on appetite, nutritional status, and quality of life in lung cancer patients: A randomized, double-blind clinical trial. Support. Care Cancer.

[64] Bar-Sela G., Zalman D., Semenysty V., Ballan E. (2019). The effects of dosage-controlled cannabis capsules on cancer-related cachexia and anorexia syndrome in advanced cancer patients: Pilot study. Integr. Cancer Ther..

[65] PDQ Supportive and Palliative Care Editorial Board (2002). Nausea and vomiting related to cancer treatment (PDQ®): Health professional version. PDQ Cancer Information Summaries.

[66] Sharkey K.A., Wiley J.W. (2016). The Role of the Endocannabinoid System in the Brain-Gut Axis. Gastroenterology.

[67] Sharkey K.A., Darmani N.A., Parker L.A. (2014). Regulation of nausea and vomiting by cannabinoids and the endocannabinoid system. Eur. J. Pharmacol..

[68] Limebeer C.L., Rock E.M., Mechoulam R., Parker L.A. (2012). The anti-nausea effects of CB1 agonists are mediated by an action at the visceral insular cortex. Br. J. Pharmacol..

[69] LiverTox: Clinical and Research Information on Drug-Induced Liver Injury (2012). Bethesda (MD): National Institute of Diabetes and Digestive and Kidney Diseases. 2012; Nabilone. https://www.ncbi.nlm.nih.gov/books/NBK547865/.

[70] Schleider L.B.-L., Mechoulam R., Lederman V., Hilou M., Lencovsky O., Betzalel O., Shbiro L., Novack V. (2018). Prospective analysis of safety and efficacy of medical cannabis in large unselected population of patients with cancer. Eur. J. Intern. Med..

[71] Vučković S., Srebro D., Vujović K.S., Vučetić Č., Prostran M. (2018). Cannabinoids and pain: New insights from old molecules. Front. Pharmacol..

[72] Abrams D.I., Jay C.A., Shade S.B., Vizoso H., Reda H., Press S., Kelly M.E., Rowbotham M.C., Petersen K.L. (2007). Cannabis in painful HIV-associated sensory neuropathy: A randomized placebo-controlled trial. Neurology.

[73] Andreae M.H., Carter G.M., Shaparin N., Suslov K., Ellis R.J., Ware M.A., Abrams D.I., Prasad H., Wilsey B., Indyk D. (2015). Inhaled cannabis for chronic neuropathic pain: A meta-analysis of individual patient data. J. Pain..

[74] Wallace M.S., Marcotte T.D., Umlauf A., Gouaux B., Atkinson J.H. (2015). Efficacy of inhaled cannabis on painful diabetic neuropathy. J. Pain..

[75] Rahn E.J., Makriyannis A., Hohmann A.G. (2007). Activation of cannabinoid CB1 and CB2 receptors suppresses neuropathic nociception evoked by the chemotherapeutic agent vincristine in rats. Br. J. Pharmacol..

[76] Khasabova I.A., Khasabov S., Paz J., Harding-Rose C., Simone D.A., Seybold V.S. (2012). Cannabinoid type-1 receptor reduces pain and neurotoxicity produced by chemotherapy. J. Neurosci..

[77] Ward S.J., McAllister S.D., Kawamura R., Murase R., Neelakantan H., Walker E.A. (2014). Cannabidiol inhibits paclitaxel-induced neuropathic pain through 5-HT(1A) receptors without diminishing nervous system function or chemotherapy efficacy. Br. J. Pharmacol..

[78] Lynch M.E., Cesar-Rittenberg P., Hohmann A.G. (2014). A double-blind, placebo-controlled, crossover pilot trial with extension using an oral mucosal cannabinoid extract for treatment of chemotherapy-induced neuropathic pain. J. Pain. Symptom Manag..

[79] Waissengrin B., Mirelman D., Pelles S., Bukstein F., Blumenthal D.T., Wolf I., Geva R. (2021). Effect of cannabis on oxaliplatin-induced peripheral neuropathy among oncology patients: A retrospective analysis. Ther. Adv. Med. Oncol..

[80] Boland E.G., Bennett M.I., Allgar V., Boland J.W. (2020). Cannabinoids for adult cancer-related pain: Systematic review and meta-analysis. BMJ Support. Palliat. Care.

[81] Abrams D.I., Couey P., Shade S.B., Kelly M.E., Benowitz N.L. (2011). Cannabinoid-opioid interaction in chronic pain. Clin. Pharmacol. Ther..

[82] Tramèr M.R., Carroll D., Campbell F.A., Reynolds D.J., Moore R.A., McQuay H.J. (2001). Cannabinoids for control of chemotherapy induced nausea and vomiting: Quantitative systematic review. BMJ.

[83] Ben Amar M. (2006). Cannabinoids in medicine: A review of their therapeutic potential. J. Ethnopharmacol..

[84] Smith L.A., Azariah F., Lavender V.T., Stoner N.S., Bettiol S. (2015). Cannabinoids for nausea and vomiting in adults with cancer receiving chemotherapy. Cochrane Database Syst. Rev..

[85] Tafelski S., Häuser W., Schäfer M. (2016). Efficacy, tolerability, and safety of cannabinoids for chemotherapy-induced nausea and vomiting—A systematic review of systematic reviews. Schmerz.

[86] Schussel V., Kenzo L., Santos A., Bueno J., Yoshimura E., Latorraca C.d.O.C., Pachito D.V., Riera R. (2018). Cannabinoids for nausea and vomiting related to chemotherapy: Overview of systematic reviews. Phytother. Res..

[87] Chow R., Valdez C., Chow N., Zhang D., Im J., Sodhi E., Lock M. (2020). Oral cannabinoid for the prophylaxis of chemotherapy-induced nausea and vomiting—A systematic review and meta-analysis. Support. Care Cancer.

[88] Hesketh P.J., Kris M.G., Basch E., Bohlke K., Barbour S.Y., Clark-Snow R.A., Danso M.A., Dennis K., Dupuis L.L., Dusetzina S.B. (2020). Antiemetics: ASCO guideline update. J. Clin. Oncol..

[89] Duran M., Pérez E., Abanades S., Vidal X., Saura C., Majem M., Arriola E., Rabanal M., Pastor A., Farré M. (2010). Preliminary efficacy and safety of an oromucosal standardized cannabis extract in chemotherapy-induced nausea and vomiting. Br. J. Clin. Pharmacol..

[90] Grimison P., Mersiades A., Kirby A., Lintzeris N., Morton R., Haber P., Olver I., Walsh A., McGregor I., Cheung Y. (2020). Oral THC:CBD cannabis extract for refractory chemotherapy-induced nausea and vomiting: A randomised, placebo-controlled, phase II crossover trial. Ann. Oncol..

[91] Zikos T.A., Nguyen L., Kamal A., Fernandez-Becker N., Regalia K., Nandwani M., Sonu I., Garcia M., Okafor P., Neshatian L. (2020). Marijuana, ondansetron, and promethazine are perceived as most effective treatments for gastrointestinal nausea. Dig. Dis. Sci..

[92] Davidson J.R., MacLean A.W., Brundage M.D., Schulze K. (2002). Sleep disturbance in cancer patients. Soc. Sci. Med..

[93] Carlini E.A., Cunha J.M., Paulo S. (1981). Hypnotic and antiepileptic effects of cannabidiol. J. Clin. Pharmacol..

[94] Betthauser K., Pilz J., Vollmer L.E. (2015). Use and effects of cannabinoids in military veterans with posttraumatic stress disorder. Am. J. Health-Syst. Pharm..

[95] Babson K.A., Bonn-Miller M.O. (2014). Sleep disturbances: Implications for cannabis use, cannabis use cessation, and cannabis use treatment. Curr. Addict. Rep..

[96] Sznitman S.R., Vulfsons S., Meiri D., Weinstein G. (2020). Medical cannabis and insomnia in older adults with chronic pain: A cross-sectional study. BMJ Support. Palliat. Care.

[97] Courts J., Maskill V., Gray A., Glue P. (2016). Signs and symptoms associated with synthetic cannabinoid toxicity: Systematic review. Australas. Psychiatry.

[98] Tait R.J., Caldicott D., Mountain D., Hill S.L., Lenton S. (2016). A systematic review of adverse events arising from the use of synthetic cannabinoids and their associated treatment. Clin. Toxicol..

[99] Goyal H., Awad H.H., Ghali J.K. (2017). Role of cannabis in cardiovascular disorders. J. Thorac. Dis..

[100] Brown J.D. (2020). Potential Adverse Drug Events with Tetrahydrocannabinol (THC) Due to Drug–Drug Interactions. J. Clin. Med..

[101] Singh A., Saluja S., Kumar A., Agrawal S., Thind M., Nanda S., Shirani J. (2018). Cardiovascular Complications of Marijuana and Related Substances: A Review. Cardiol. Ther..

[102] Siegel R.L., Giaquinto A.N., Jemal A. (2024). Cancer statistics, 2024. CA Cancer J. Clin..

[103] Senga S.S., Grose R.P. (2021). Hallmarks of cancer—The new testament. Open Biol..

[104] Aggarwal S. (2016). Use of Cannabinoids in Cancer Care: Palliative Care. Curr. Oncol..

